# CEP-MI: a novel cue-execution dual-stage motor imagery paradigm with PGCANet decoding architecture

**DOI:** 10.3389/fnhum.2026.1908608

**Published:** 2026-10-22

**Authors:** Jianxiu Li, Pengda Yu, Huiyu Qiao, Zhiqiang Zheng, Changming Wang, Zhi Weng, Caili Gong

**Affiliations:** 1 School of Electronic Information Engineering, Inner Mongolia University, Hohhot, China; 2 Key Laboratory of Intelligent Communication and Sensing and Signal Processing, Hohhot, China; 3 Inner Mongolia Vocational and Technical College of Transportation, Chifeng, China; 4 School of Biomedical Engineering, Capital Medical University, Beijing, China

**Keywords:** brain-computer interface, CEP-MI paradigm, desynchronization, event-related potential, multi-domain fusion, synchronization

## Abstract

**Background:**

Motor imagery-based brain-computer interface (MI-BCI) holds great potential in neurorehabilitation and motor assistance. Data quality and motor intention decoding effectiveness are closely associated with participants’ engagement. However, previous MI tasks ignored active engagement regulation, and neural mechanisms underlying the transition from motor imagery to execution remain unclear, with limited relevant research. Conventional separate-trial designs cannot capture continuous brain dynamics from imagery to execution and cannot objectively evaluate subject engagement, which hinders mechanism exploration and stable decoding.

**Methods:**

To address these paradigm-related limitations, a novel cue-execution MI paradigm (CEP-MI) with countdown anchors was designed to quantify engagement. Motor execution sessions were added post-imagery to reveal neural differences during the transition and identify movement-related brain activities. Meanwhile, to boost MI-EEG decoding performance, a collaborative decoding architecture (PGCANet) integrating prior guidance and channel awareness was proposed, with dual branches and consistency constraint loss for decision fusion.

**Results:**

CEP-MI effectively quantifies participant engagement, and PGCANet achieves superior decoding performance compared to traditional algorithms and advanced models, with the optimal average accuracy reaching 85.77% in binary classification and 82.47% in quadruple classification.

**Conclusion:**

Targeting the above-mentioned research gaps, the CEP-MI paradigm provides valuable insights into the imagery-execution transition mechanism. PGCANet enhances MI decoding accuracy via multi-branch collaboration, laying a foundation for MI-BCI’s clinical application in neurorehabilitation.

**Significance:**

This study introduces a novel paradigm and collaborative architecture that significantly advance MI-BCI decoding accuracy and engagement quantification, and helps to promote its clinical application in neurorehabilitation.

## 1 Introduction

Movement is an essential activity for sustaining normal human life and work, involving key stages such as preparation and execution (Fu et al., 2025; Gwon and Ahn, 2024; Martinez-Peon et al., 2024; Tantawanich et al., 2024). In recent years, closedloop motor imagery-based brain computer interface (MI-BCI) technology has shown considerable promise in promoting motor recovery (Lim et al., 2026; Lin et al., 2026; Song et al., 2025; Takasaki et al., 2026). Non-invasive electroencephalography (EEG) is widely used in BCI development due to its avoidance of surgical risks and invasiveness.

Neural activity in the brain exhibits rhythmic fluctuations within specific frequency bands. Event-related desynchronization (ERD) and synchronization (ERS) occur in the *μ* (8–13 Hz) and β (14–30 Hz) rhythms over the mot or cortex (Pfurtscheller et al., 2006; Wen et al., 2024), reflecting the generation and modulation of motor intention. Studies have demonstrated that brain region activation in motor execution (ME) and MI is similar during early stages, but exhibits significant differences between the motor response stage of ME and the imagination maintenance stage of MI (Borràs et al., 2025; Moran et al., 2012; Jeannerod, 1994). MI is associated with motor planning brain regions, characterized by contralateral ERD activation and ipsilateral ERS inhibition, with limited activation in the MI cortex (Duann and Chiou, 2016; Jeon et al., 2011). In contrast, ME involves actual motor output, resulting in greater activation of motor cortex regions dominated by MI and more intense ERD responses (Jeon et al., 2011; Wang et al., 2026; Kraeutner et al., 2014). These distinct neural patterns provide the physiological basis for MI-BCI design, yet effectively capturing them depends critically on how the motor imagery task is structured and presented to users.

A variety of MI-BCI paradigms have been developed to decode motor intention from EEG signals, yet these designs still have notable limitations. Therefore, constructing an effective MI paradigm is key to ensuring reliable decoding results enabling exploration of neural mechanisms. Most existing paradigms separate MI and ME into independent tasks and cannot capture their intermediate transition states; hence a dedicated paradigm is required to characterize the continuous neural dynamics across MI to ME conversion. In recent years, paradigm design has gradually shifted from single modality to multimodal fusion. For instance, introducing visual and spatiotemporal tactile synchronized stimulation can enhance sensorimotor cortex activation in subjects with poor performance (Yin et al., 2025), while action sequence guidance paradigms have been shown to effectively improve both offline and online decoding accuracy (Kong et al., 2025). A paradigm combining motor imagery with electrical muscle stimulation has been used to systematically distinguish the EEG characteristics of motor errors, stimulus-driven attention, and cognitive conditions, revealing that mu-band suppression reflects the consistency between prediction and sensory input (Suemitsu and Nambu, 2026). Additionally, the preparatory movement state paradigm can induce significant energy changes during the movement preparation stage, triggering ERD earlier than traditional paradigms and achieving high classification accuracy through multi-stage feature fusion (Zhang et al., 2026). Studies have also shown that feedback patterns during BCI training directly influence cortical activation; for example, paradigms without false positive feedback induce higher cortical activation in the contralateral motor area (Jeong et al., 2025). Moreover, advanced decoding methods such as Riemann tangent space mapping and cross-frequency coupling can further enhance the classification accuracy of MI-EEG in multi-class tasks, demonstrating that the combination of paradigm design and algorithmic innovation helps improve the reliability of decoding (Xiong et al., 2025; Tai et al., 2024).

Despite these advances, most existing paradigms cannot capture the transitional neural states between MI and ME, leaving an important research gap. Current research predominantly focuses on independent MI or ME processes, with limited exploration of neural processes during state transitions. Competitions and research papers related to BCI have introduced various MI paradigms (Miao and Zhao, 2024; Wang et al., 2024; Wang et al., 2023). The PhysioNet dataset paradigm enables subjects to conduct independent MI and ME experiments (Goldberger et al., 2000), providing a foundation for comparative studies of these movement patterns. According to motor cognition theory (Jeannerod, 2006), MI represents a continuous process ranging from pure mental representation at one end to actual movement execution at the other, with transitional processes bridging the two states. When abnormal neural signals, such as delayed motor cortex activation, occur during transitional stages, these signals can serve as precise therapeutic targets for rehabilitation intervention. Furthermore, neural signal intensity during transitional stages can function as a key indicator for evaluating motor function recovery progress (Pomeroy et al., 2011). Consequently studying the conversion process from MI to ME process can not only elucidate collaborative mechanisms among brain regions during motor control but also provide more precise guidance for clinical rehabilitation practice.

Beyond paradigm design, subject participation represents another crucial prerequisite for effective MI decoding. Most existing MI tasks treat participation as a default stable variable without implementing quantification mechanisms. We often observe data quality fluctuations among subjects due to distraction or failure to imagine designated actions as required by experimental protocols. For instance, in the BCI IV-2A dataset classification task (Brunner et al., 2008), when identical algorithms were applied, classification accuracy for subjects with IDs 2 and 6 was significantly lower than that of other subjects. This substantial accuracy difference underscores the necessity of establishing participation quantification mechanisms, providing an important foundation for subsequent design of targeted participation assessment.

To improve decoding robustness despite data quality fluctuations, effective feature representation becomes essential. Many previous studies have attempted to fuse frequency-domain features of EEG with spatial–temporal features. Ai et al. (2025) proposed a holographic convolutional attention neural network that integrates spatial–temporal-frequency features. FBCNet (Mane et al., 2021) employs multi-view data representations to extract discriminative features in the frequency-spatial domain and introduces a novel variance layer to effectively aggregate temporal information from EEG signals. TSFFNet (Miao and Zhao, 2024) integrates EEG signal features at the feature level, using continuous wavelet transform (CWT) for frequency domain extraction and dedicated temporal and spatial convolution modules for capturing temporal and spatial domain features. However, traditional methods are highly dependent on data quality and distribution, or require coverage of all electrode channels, introducing redundant information and increasing computational complexity.

To address the above limitations, this work constructs a new experimental paradigm and decoding framework for exploring MI to ME neural transition. Aiming to improve the accuracy of EEG signals classification, in this study, a novel EEG dataset was collected from 97 participants using a psychology-informed experimental paradigm, a task design guided by motor cognition principles that employs countdown anchors and continuous MI-to-ME task sequences to conform to natural human motor cognitive processes, that integrates MI with motor execution and incorporates countdown anchors to elicit event-related potentials. This paradigm enables both objective assessment of participant engagement and analysis of neurophysiological changes during the MI-to-ME transition in the time and frequency domains. Additionally, a collaborative decoding framework with prior guidance and channel-aware enhancement, termed PGCANet, has been proposed to address feature fragmentation.

The remainder of this paper is organized as follows: section II introduces the architecture of the proposed algorithm. Section III describes the proposed MI experimental paradigm. Section IV presents the experimental results and an interpretability analysis. Section V discusses the findings and research contributions. Finally, Section VI summarizes the study and concludes with the key takeaways.

## 2 Experimental analysis

### 2.1 Subjects

Ninety-seven healthy subjects (80 males, 94 right-handed, aged 18–28 years, mean 23.6 ± 1.16) participated in the MI experiment. Prior to the experiment, all subjects were provided with detailed information regarding the experimental procedure and signed a written consent in accordance with the Declaration of Helsinki. Exclusion criteria were having a history of neurological or musculoskeletal disorders. The study was approved by the Institutional Review Board of Inner Mongolia University People’s Hospital. Participant demographic characteristics are summarized in Table 1.

**Table 1:** Demographic characteristics of the participants.

Characteristic	Value
Right handed: Left handed	94: 3
Age	23.6 ± 1.16
Age range	18–28
Male	80
Female	17

### 2.2 Cue-execution dual-stage MI paradigm

Existing hybrid MI-ME paradigms mostly record MI and ME in separate trials, enabling only offline comparisons between MI and ME EEG features. They are incapable of characterizing the continuous neural transition from imagery to execution. By contrast, sequential motor-imagery paradigms involve purely mental imagery without actual limb output. Distinct from these prior schemes, the proposed CEP-MI paradigm constructs a continuous transition from MI to ME within a single trial. Meanwhile, it introduces countdown anchors to record EEG responses during the imagery-execution transition phase, yielding methodological innovations that differentiate it from existing paradigms.

Fatigue or inattention may impair performance, but traditional paradigms cannot assess concentration during long experiments. Therefore, we added a digital countdown above the arrow to analyze ERPs as an attention measure. Additionally, to explore the neural transition between motor imagery and motor execution, we introduced an ME stage after the traditional MI. Each participant performed four MI tasks (left/right hand, left/right foot) in a pseudo-randomized order. Figure 1 shows the trial timing of the paradigm.

**Figure 1:**
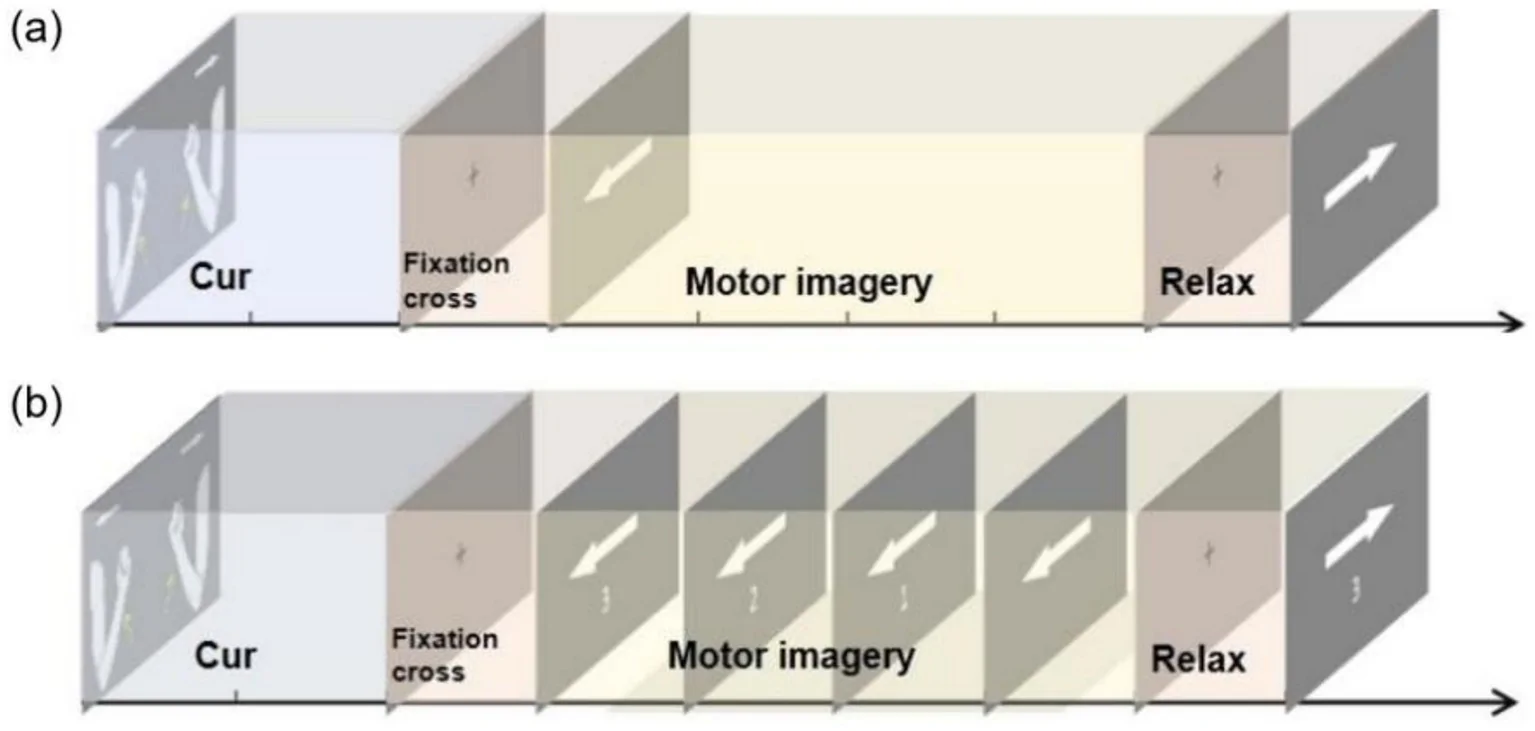
Comparison of single-trial timing between conventional and proposed motor imagery paradigms. **(a)** Classic arrow-cued motor imagery paradigm; **(b)** The proposed CEP-MI dual-stage paradigm, featuring countdown anchors and continuous transition from motor imagery to motor execution within one trial.

As shown in Figure 1a, each trial of the classic arrow paradigm began with a 1 s fixation cross on a black screen, followed by a left- or right-pointing arrow cue for 1 s. Participants then had 4 s to imagine moving the corresponding limb, followed by a 1 s black-screen break. Figure 1b illustrated the timeline of the cue-execution MI task. A fixation cross appeared on a black screen for 1 s. Then, a countdown arrow (3, 2, 1) was shown. During the countdown, participants performed motor imagery. After the countdown, they made a slight limb movement together with MI, followed by a 1 s rest.

Each experimental paradigm comprised two runs. Run 1 followed the classic MI paradigm with 48 trials (12 per MI task). Run 2 involved a ME task with 68 trials (17 per MI task). The order of the two runs was counterbalanced across participants. Participants were instructed to maintain physical stability throughout the session and to avoid actions such as swallowing, in order to improve the quality of the collected data.

### 2.3 Data acquisition and preprocessing

EEG was recorded at a sampling rate of 1,000 Hz with a 64-channel Neuroscan system. The scalp impedance of each sensor was kept below 10 kΩ and EEG data were re-referenced off-line to the average of all electrodes.

Offline preprocessing of EEG data was performed using EEGLAB software.^1^ The data were down-sampled to 250 Hz, and eye blinks were removed using independent component analysis. Subsequently, the signal was bandpass filtered with a high-pass cutoff of 4 Hz and a low-pass cutoff of 45 Hz. After excluding non-scalp electrodes, EEG data from a total of 62 electrodes were retained, resulting in the CEP-MI dataset comprising 97 healthy subjects for subsequent analysis.

### 2.4 Datasets

The proposed experiment was evaluated on both our private dataset and a widely used public dataset, BCIC-IV-2a (Brunner et al., 2008). This public dataset consists of EEG recordings from nine healthy subjects (A01–A09), acquired using 22 Ag/AgCl electrodes at a sampling rate of 250 Hz. Each participant completed two sessions (training and evaluation), with 288 trials per session, and each motor imagery task was repeated 72 times per session. For analysis, the 2–6 s time window was selected as the trial sample.

## 3 Proposed methodology

The proposed PGCANet consists of two branches and four primary components: channel-aware enhanced spatial–temporal feature extraction, time-frequency representation, priori guided time-frequency feature extraction, and decision-level feature fusion based on consistency-constrained fusion loss. The architecture is illustrated in Figure 2.

**Figure 2:**
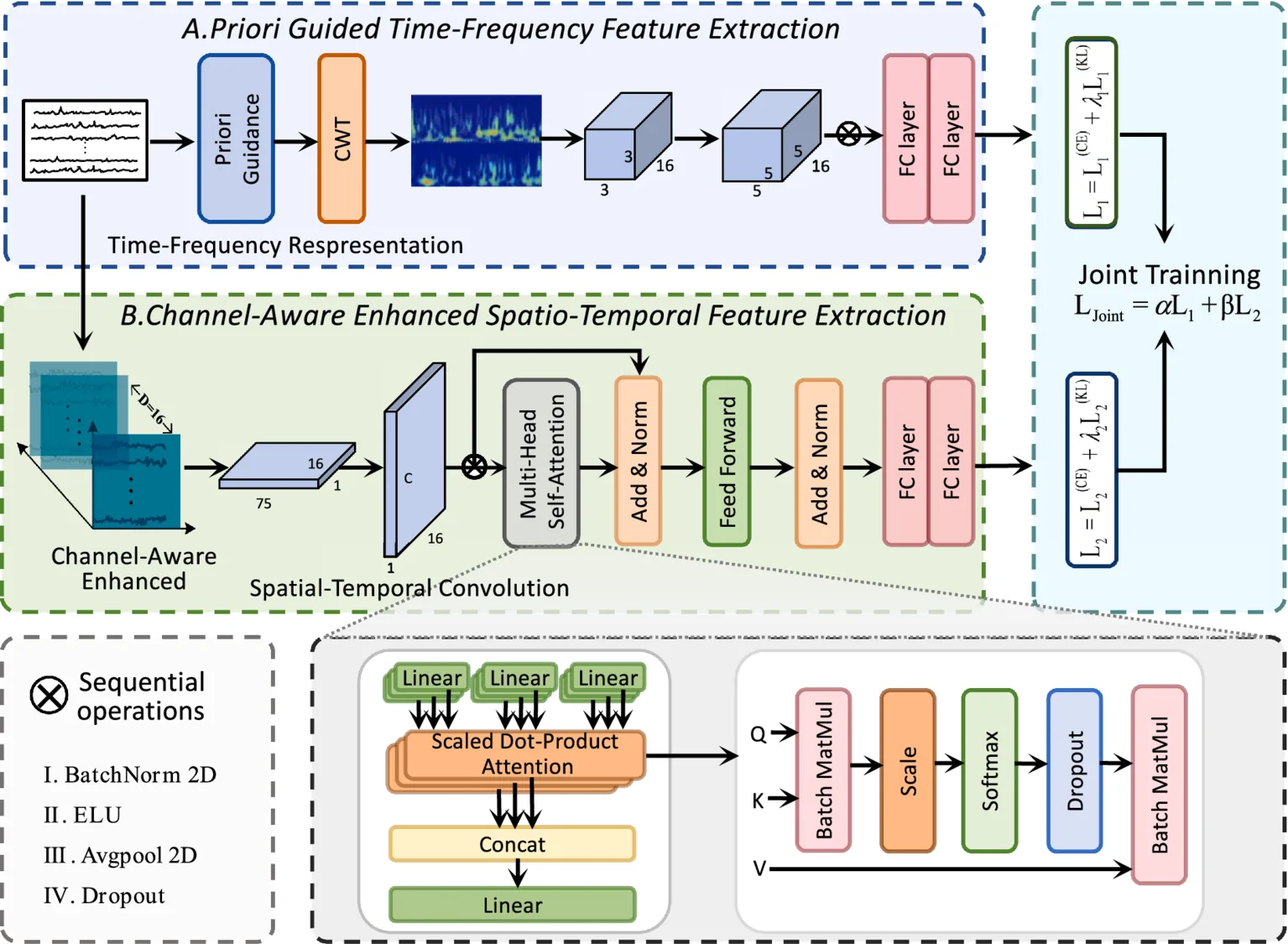
Architecture of the proposed PGCANet for MI EEG decoding. It has two parallel branches: **(A)** priori-guided time-frequency extraction via continuous wavelet transform, and **(B)** channel-aware spatio-temporal extraction with multi-head self-attention, optimized by a joint loss function.

In the channel-aware enhanced spatial–temporal feature extraction branch, channel-aware enhanced module encodes spatial information into the depth dimension. Spatial–temporal convolution and attention mechanism capture the spatial correlations and temporal features between electrodes. The time-frequency feature extraction branch takes time-frequency maps as input. Leveraging prior knowledge, it precisely extracts energy patterns related to MI. The two branches are trained collaboratively through consistency constraint fusion loss. The network processes appropriately preprocessed EEG data as input. A detailed description of the network architecture is provided below.

### 3.1 Channel-aware enhanced spatial–temporal feature extraction

The combination of temporal and spatial convolutions has been widely adopted in EEG MI decoding (Song et al., 2023). However, this architecture does not fully integrate spatial information into the temporal convolution layers. This limitation restricts the model’s ability to capture spatial dependencies effectively within EEG data. To overcome this limitation and improve the model’s capacity to learn spatial features, we propose a channel-aware enhanced module. This module assigns learnable parameters to each channel, which are continuously updated during training. This allows the network to prioritize the most informative channels.

The structure of our spatial–temporal feature extraction network is as follows: the input data has a shape of 1 × *C* × *T*, where *C* denotes number of channels and T represents number of time points. Data first passes through the channel-aware enhanced module, which performs tensor product operations. This is followed by the temporal convolution module with a depth of 16 and a kernel size of 1 × 25 to extract temporal features. Then it is processed by the spatial convolution module with a depth of 16 and a kernel size of *C* × 1 to capture spatial features. Finally, a multi-head self-attention mechanism models long-term dependencies, and the output is classified through two fully connected layers.

To tackle the spatiotemporal modeling limitations of conventional EEG decoding methods, the proposed channel-aware architecture performs spatial convolution over multi-channel EEG signals combined with temporal modeling. It adaptively learns channel-wise weights to quantify the task-related information of each electrode. Specifically, the architecture prioritizes the most informative channels by generating learnable channel attention weights. Channels carrying strong MI-related neural signals are assigned larger weights, while weights for less informative or noisy channels are suppressed during feature extraction.

The design of the channel-aware enhanced module is inspired by signal source reconstruction techniques. This module processes the input data by extending its spatial information into the depth dimension through tensor multiplication operations. Let *x* ∈ R 1 × *C* × *T* represent an input EEG sample, where *C* denotes the number of channels and *T* indicates the number of time sampling points. Let c ∈ R *D* × 1 × *C* represent a tensor following a normal distribution, where *D* corresponds to the number of instances, which equals the number of convolutional kernels. The computational process of the channel-aware enhanced module is illustrated in Equation 1:

$$X_{\mathrm{hct}}^{,}=\sum_{d}X_{\mathrm{dct}}C_{\mathrm{hdc}}$$ (1)

The subscript in the formula indicates the dimension of the corresponding tensor. In summary, the channel-aware enhanced module effectively transfers spatial feature information to the depth dimension while maintaining low computational complexity and using fewer parameters. This approach integrates channel information with subsequent temporal convolutions. This integration mitigates feature fragmentation caused by the dispersed spatial distribution and complex temporal features of EEG signals. As a result, the module enhances the network’s ability to focus on distinct channel features. We adopt the multi-head self-attention structure to capture diverse, multi-scale spatio-temporal dependencies within EEG signals. Inspired by Transformer, we only introduce the multi-head attention component rather than the full Transformer architecture to reduce computational overhead and avoid overfitting on limited MI EEG datasets.

### 3.2 Time-frequency mapping construction via continuous wavelet transform

Research has shown that in three-channel MI tasks (Miao and Zhao, 2024; Chen et al., 2026; Wei et al., 2024), EEG data obtained exclusively from the C3, C4, and Cz electrodes yields superior classification performance. These electrodes exhibit the strongest association with motor rhythms. Building on this finding, we hypothesized that classification performance in multi-channel EEG data could be similarly enhanced by selecting only the electrodes with the most prominent time-frequency features. In this study, leveraging prior knowledge, we focused on the C3 and C4 electrodes. We applied CWT to convert the time-domain EEG signals from these channels into time-frequency representations. The Morlet wavelet is selected as the mother wavelet for the transformation in this study. Compared with other wavelet bases, the Morlet wavelet achieves balanced time-frequency localization performance. It can effectively characterize the oscillatory μ and β sensorimotor rhythms that dominate motor-imagery EEG, which are the core discriminative features for our decoding task. The operational process of the CWT is described in Equation 2:

$$\mathrm{CWT}\;\;(a,b)=\frac{1}{\sqrt{a}}\int s(t)\Psi (\frac{t-b}{a})\mathrm{dt}$$ (2)

In this context, *ψ* denotes the mother wavelet function, while a and b represent the scale and shift parameters, respectively. This study utilizes the Morlet wavelet as the mother wavelet function, which is defined as shown in Equation 3.

$$\psi (t)=\exp(-\frac{\beta^{2}t^{2}}{2})\cos(\mathrm{\pi t})$$ (3)

### 3.3 Priori guided time-frequency feature extraction

In this study, the prior knowledge refers to the well-documented lateralized sensorimotor rhythm modulation at C3 and C4 electrodes during motor imagery, where ERD/ERS effects supply discriminative information for left/right MI classification. This knowledge is integrated into PGCANet through the prior guidance module to extract task-related EEG features from these key channels.

After obtaining the time-frequency maps of the two-channel EEG signals, we designed a shallow CNN to extract abstract features from the selected channels. To capture features at different scales, we used two sets of convolutional kernels: 16 kernels of size 3 × 3 and 16 kernels of size 5 × 5. The last convolutional layer is followed by a series of operations, including average pooling, batch normalization, and dropout. These operations serve to reduce the dimensionality of the data while preserving essential feature information and preventing overfitting.

We use the exponential linear unit as the activation function to handle multidimensional signals and capture complex EEG patterns (e.g., low- and high-frequency fluctuations), enabling detailed feature extraction and improving nonlinear learning. Two fully connected linear layers then produce the final classification output.

### 3.4 Decision-level feature fusion based on consistency-constrained fusion loss

To effectively leverage the multi-domain characteristics of EEG data, several studies have explored data fusion at the feature level by directly concatenating flattened feature vectors from different domains (Huang et al., 2025; Wang et al., 2025). However, this approach has notable limitations. Flattening feature vectors results in the loss of inherent structural information present in the original data. This significantly compromises the integrity of the extracted features. Dropout, a widely used form of stochastic regularization, has a key characteristic. When the same input undergoes two independent dropout operations, the resulting outputs consistently differ, yet remain dependent. Building on this property, we devised a consistency-constrained fusion loss.

The operational logic of this loss function is described below. First, the same input sample undergoes two independent dropout operations sequentially. Next, the Kullback–Leibler divergence (Joyce, 2011) is introduced as a constraint term to regulate the predictions from both operations. This ensures a degree of similarity between the two predictions while maintaining subtle differences. The constraint mechanism directs the model to capture core data representations more stably. This prevents excessive reliance on local features.

Specifically, let the input data for the prior-guided spatial–temporal feature extraction branch be *x_f_*, and the input data for the channel-aware enhanced spatial–temporal *x_t_*. We perform two forward passes through the network for each branch’s input data, yielding two sets of prediction distributions $P_{1}^{w}(y_{f}\;|x_{f})$ , $P_{2}^{w}(y_{f}\;|x_{f})$ , $P_{1}^{\theta }(y_{t}\;|x_{t})$ , $P_{2}^{\theta }(y_{t}\;|x_{t})$ . The consistency constraint fusion loss, comprising cross-entropy and Kullback–Leibler divergence components as shown in Equations 4–6 is applied to both branches. Here, λ is a tunable parameter set to 0.02 in this study.

$$L_{1}^{\left(\mathrm{CG}\right)}=-\log F_{1}^{w}(y_{f}|x_{f})-\log F_{2}^{w}(y_{f}|x_{f})$$ (4)

$$\begin{array}{l} L_{1}^{\left(\mathrm{KL}\right)}=\frac{1}{2}(D_{\mathrm{KL}}(F_{1}^{W}(y_{f}\;|\;x_{f})\;\|P_{2}^{W}(y_{f}\;\;|\;x_{f})))\\ +D_{\mathrm{XY}}(P_{2}^{W}(y_{f}\;|\;x_{f})\|\;P_{1}^{W}(y_{f}|\;x_{f})) \end{array}$$ (5)

$$L_{1}=L_{1}^{\left(\mathrm{CE}\right)}+\lambda_{1}L_{1}^{\left(\mathrm{KL}\right)}$$ (6)

Similarly, the loss functions for the channel-aware enhanced spatial–temporal feature extraction branch are presented in Equations 7–9.

$$L_{2}^{\left(\mathrm{CF}\right)}=-\log P_{1}^{\theta }(y_{t}\;|\;x_{i})-\log P_{2}^{\theta }(y_{t}\;|\;x_{i})$$ (7)

$$\begin{array}{l} L_{2}^{\left(\mathrm{XL}\right)}=\frac{1}{2}(D_{\mathrm{XL}}(P_{1}^{\theta }(y_{t}|\;x_{t})\;\|\;P_{2}^{\theta }(y_{t}|\;x_{t}))\\ +D_{\mathrm{XL}}(P_{2}^{\theta }(y_{t}\;|\;x_{t})\;\|P_{1}^{\theta }(y_{t}|\;x_{t})) \end{array}$$ (8)

$${\bar{L}}_{2}=L_{2}^{\left(G\right)}+\lambda_{2}L_{2}^{\left(\mathrm{KL}\right)}$$ (9)

The final joint training loss function is a weighted combination of the two branch loss functions, as presented in Equation 10. In this study, both parameters *α* and *β* are set to 0.5:

$$L_{\text{jomt}}=\alpha L_{1}+\beta L_{2}$$ (10)

### 3.5 Experimental setup

The PGCANet model was trained using the PyTorch deep learning framework. For the training configuration, the Adam optimizer was utilized with an initial learning rate of 2 *×* 10^*−*4^. A learning rate warm-up strategy with *T*0 = 50 was employed to mitigate parameter oscillations caused by excessively high learning rates during the early training phase. This strategy ensures a smoother transition to stable training conditions and facilitates improved model convergence. The model underwent training for 2000 epochs, employing the proposed consistency-constrained fusion loss function and 0.1 label smoothing to enhance generalization and robustness.

To accommodate the diverse characteristics of the datasets, the batch size was set to 32. A universal dropout rate of 0.5 was applied at the architectural level to prevent overfitting. All datasets were partitioned into training and testing sets at a 1:1 ratio. These measures collectively ensured the stability and high performance of the model in EEG signal classification tasks.

### 3.6 Baseline comparison

To conduct a comprehensive baseline comparison in BCI decoding, we systematically evaluated representative algorithms. The classical Common Space Pattern (CSP) (Müller-Gerking et al., 1999) served as the foundational baseline, while Filter Bank CSP (FBCSP) (Ang et al., 2008) enhances discrimination via band-specific feature extraction. Among deep learning approaches, we included EEGNet (Lawhern et al., 2018), a compact yet effective CNN architecture. FBCNet (Mane et al., 2021), which combines FBCSP’s multi-band paradigm with deep neural networks to boost representational capacity. For hybrid architectures, we incorporated EEG Conformer (Song et al., 2023), which synergistically integrates CNN-based local feature extraction with Transformerbased global dependency modeling. Finally, we assessed the Flashlight-Net (Dang et al., 2024), whosenovel modular design and cross-dataset transfer learning framework performance over previous models. This progression from traditional to contemporary methods provides a rigorous evaluation framework.

## 4 Experimental results

### 4.1 Event-related potential results

To illustrate differences among left hand, right hand, left foot, and right foot in both traditional and novel tasks, ERPs were extracted following stimulus onset and plotted in Figure 3. Analyses focused on the N1 and P2 components at channel Oz, as well as the P3 and late positive component (LPC) at channel Pz.

**Figure 3:**
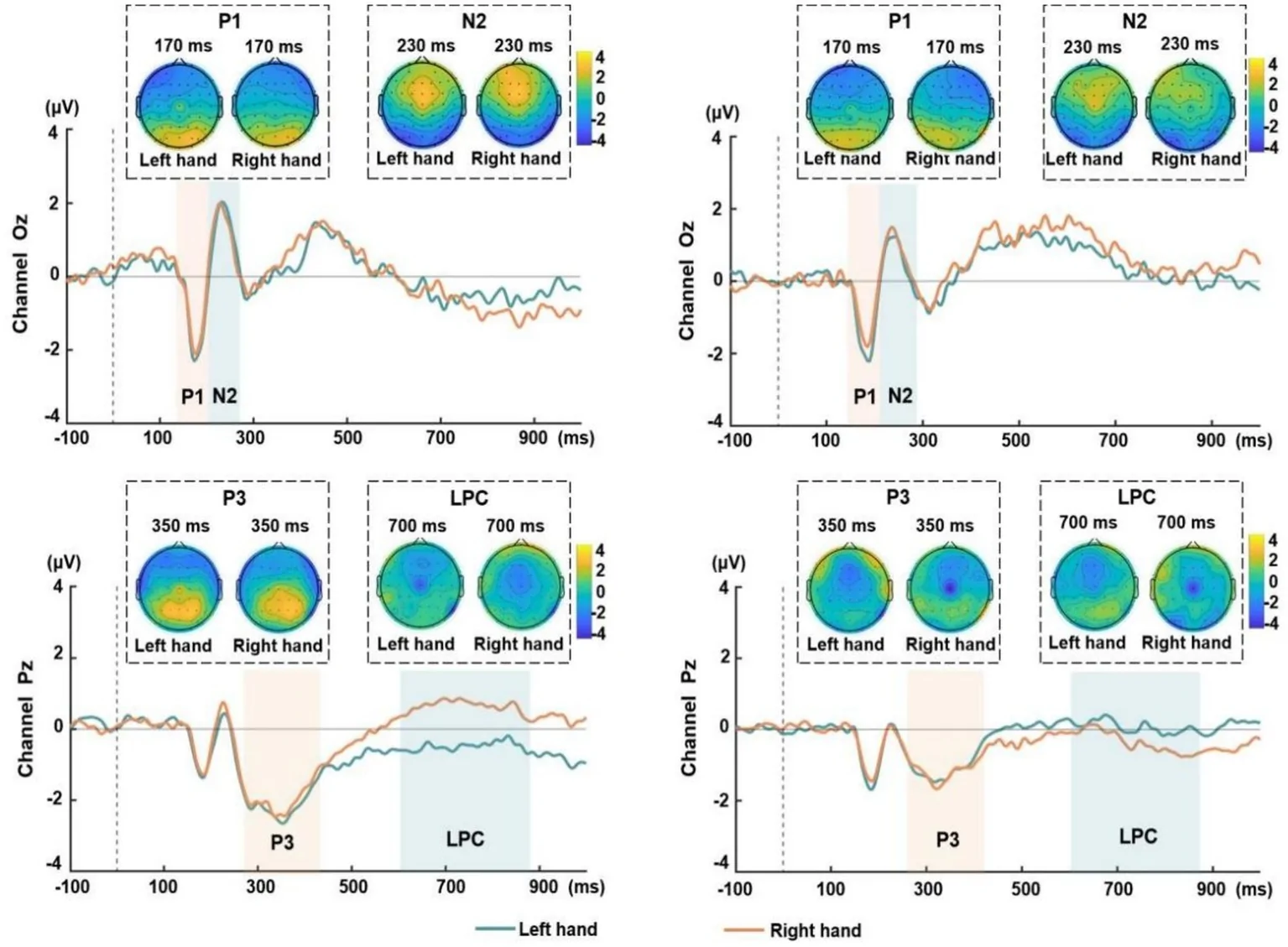
ERPs analysis following stimulus onset, with emphasis on N1 and P2 at channel Oz, and P300 with LPC at channel Pz.

For ERP amplitudes, a repeated-measures ANOVA was performed using SPSS, with task (conventional vs. novel) and limb type (left vs. right) as within-subject factors, separately for hand and foot. The Greenhouse–Geisser correction was applied for non-sphericity. Partial eta squared ( $\eta_{p}^{2}$ ) was reported as the effect size for all ANOVA effects.

Statistical analysis of P3 amplitude revealed a significant main effect of task (F1,96 = 12.65, *p* = 0.001, $\eta_{p}^{2}$ =0.116), indicating higher P3 amplitude in the cue-execution MI task (3.95 ± 0.28 μV) than the classic MI task (2.91 ± 0.23 μV), as shown in Figure 3. Additionally, a significant task × limb type interaction (F1,96 = 4.54, *p* = 0.036, $\eta_{p}^{2}$ = 0.045) was observed at electrode Pz for LPC amplitude. For the two-way interaction, simple-simple effect analyses were performed. We found that in classic MI task, right hand trials (1.93 ± 3.29 μV) had more positive LPC amplitude relative to left hand trials (0.80 ± 4.04 μV, F1,96 = 5.44, *p* = 0.022, $\eta_{p}^{2}$ =0.054). When imagining right hand movement, the cue-execution MI task although the difference in LPC amplitude between the cue-execution (1.76 ± 3.49 μV) and classic MI tasks (1.93 ± 3.29 μV) for the right hand did not reach statistical significance (F1,96 = 3.13, *p* = 0.08, $\eta_{p}^{2}$ =0.032), the numerical trend toward lower amplitude in the cue-execution task warrants further investigation with a larger sample size. No significant main or interaction effects were found for N1 or P2 amplitudes.

### 4.2 ERS and ERD results

We performed a time-frequency analysis to examine ERS and ERD patterns during MI tasks. Specifically, we processed EEG data from the C3, C4 and Cz electrodes of all 97 subjects using an 8–30 Hz bandpass filter. For each trial, the filtered signals were squared and then averaged across trials for each corresponding task condition. Meanwhile, we applied a sliding time window technique to smooth the averaged time-frequency curves (Sartipi and Cetin, 2024).

As shown in Figures 4, 5, for classical MI tasks, significant *μ*/*β* rhythm ERD occurs at the contralateral electrode C3, C4 and Cz, reaching its lowest point approximately at 0.5–1.5 s; followed by lateralization at C3/C4 and shows no statistically significant differences, which is consistent with the scalp mapping where the foot area is closer to the midline Cz. We preliminarily verified key physiological features in this dataset. It is worth noting that for tasks involving ME, hand imagery activities induced stronger resynchronization at 2–3 s, while left and right foot activities showed statistically significant differences at C3 around 3–4 s and at C4 around 1 s resynchronization (beta rebound, ERS at 2–3 s). In contrast, foot MI does not exhibit strong.

**Figure 4:**
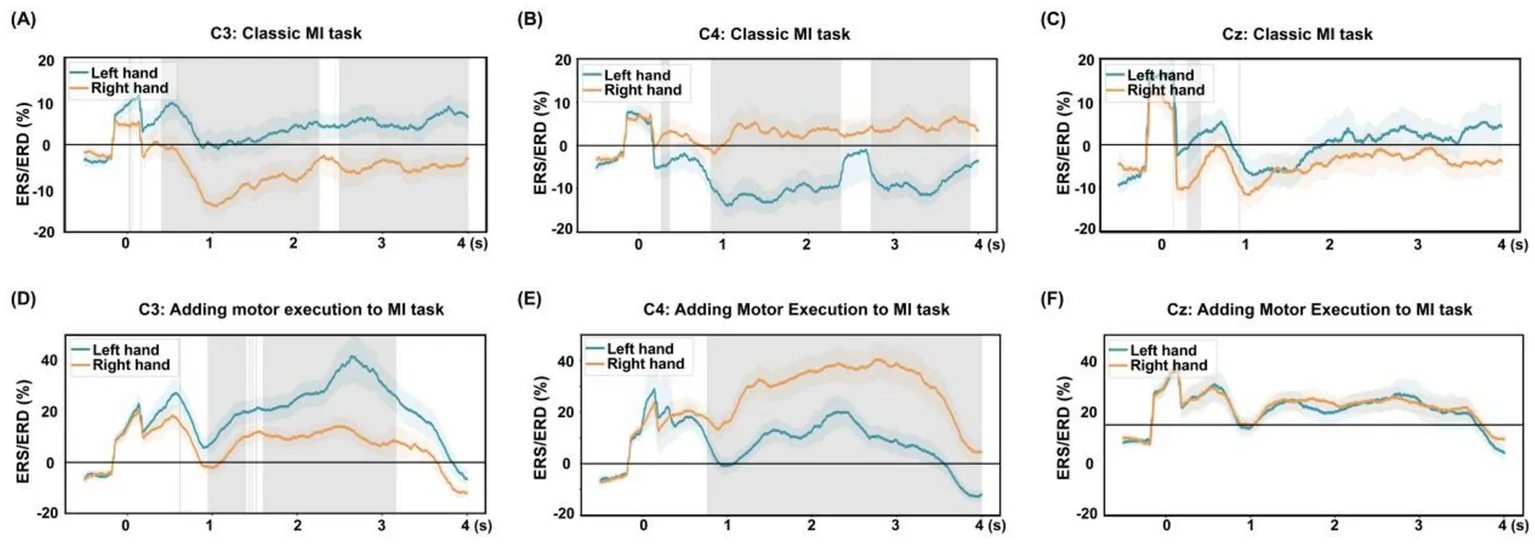
ERD/ERS on the C3, C4 and Cz electrodes during hand motor imagery.

**Figure 5:**
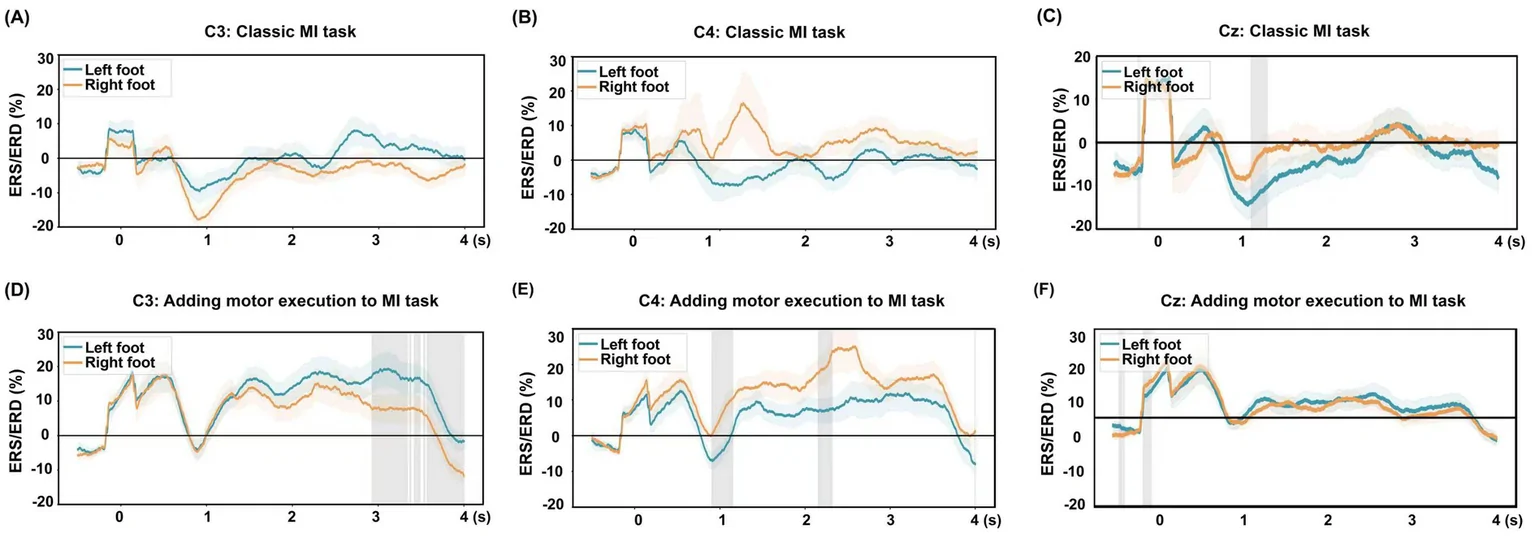
ERD/ERS on the C3, C4 and Cz electrodes during foot motor imagery.

### 4.3 Classification performance comparison

To verify whether the proposed CEP-MI paradigm enhances neural feature discriminability of EEG signals, we conducted a cross-paradigm comparison experiment using our self-built dataset of 97 subjects. As is shown in the Tables 2, 3, the CEP-MI paradigm achieves 85.77% accuracy for hand MI and 82.06% for foot MI, compared to 80.82 and 80.00% under the classical MI paradigm. A paired comparison by participant revealed that the improvements on the hand MI task were statistically significant across all five classifiers (all *p* < 0.05), with the largest gain for PGCANet (+4.95%, *p* < 0.001). On the foot MI task, the improvements were generally smaller; only PGCANet reached nominal significance (+2.06%, *p* = 0.017). This pattern suggests that the CEP-MI paradigm yields more reliable decoding gains for hand MI, likely due to the stronger lateralized sensorimotor activation elicited by hand imagery.

**Table 2:** Upper extremity motor imagery binary classification.

Method	Classic MI	CEP_MI	Δ(%)	*p* value
CSP	54.06 ± 0.13	60.34 ± 13.49	+6.28	0.00256
FBCSP	57.05 ± 10.85	61.34 ± 9.75v	+4.29	0.0036
EEGNet	70.72 ± 10.29	75.05 ± 12.38	+4.33	0.0040
Conformer	77.73 ± 10.24	80.41 ± 7.58	+2.68	0.019
PGCANet(ours)	80.82 ± 11.37	85.77 ± 6.49	+4.95	0.0048

**Table 3:** Lower extremity motor imagery binary classification.

Method	Classic MI	CEP_MI	Δ(%)	*p* value
CSP	50.93 ± 13.42	53.58 ± 12.52	+2.65	0.193
FBCSP	54.56 ± 18.04	54.19 ± 12.67	−0.17	0.910
EEGNet	68.66 ± 11.08	71.34 ± 9.76	+2.68	0.079
Conformer	76.70 ± 13.28	78.35 ± 12.55	+1.65	0.320
PGCANet(ours)	80.00 ± 8.21	82.06 ± 8.26	+2.06	0.017

To verify the effectiveness of the proposed PGCANet classification framework, tests were conducted on two datasets. As is shown in Table 4, PGCANet is superior to the comparison methods. The average classification accuracy on the public dataset BCI IV-2A reached 82.47%, verifying the excellent classification performance of PGCANet and its robust performance in cross-dataset scenarios. To further assess the practicality of PGCANet, we analyze its computational complexity. The proposed model contains only 0.73 M parameters (2.9 MB in FP32) and requires approximately 128.2 MFLOPs for a single forward inference, demonstrating a compact architecture with high inference efficiency.

**Table 4:** Quadruple classification results of different algorithms on BCI IV-2A.

Model	A01	A02	A03	A04	A05	A06	A07	A08	A09	Avg	Std
FBCSP	77.78	55.56	79.51	63.19	53.47	46.88	86.81	81.25	68.06	68.06	14.11
EEGNet	85.76	61.46	88.54	67.01	55.90	52.08	89.58	83.33	86.81	74.50	14.36
FBCNet	85.42	60.42	90.63	76.39	74.31	53.82	84.38	79.51	80.90	76.20	11.97
Conformer	88.19	61.46	93.40	78.13	52.08	65.28	92.36	88.19	88.89	78.66	14.43
Flashlight-net	89.03	**66.87**	93.82	**84.93**	62.85	**71.46**	91.77	86.32	84.06	81.23	10.63
PGCANet(ours)	**92.24**	63.79	**93.97**	68.97	**82.76**	64.66	**93.10**	**91.38**	**91.38**	**82.47**	12.24

Bold values represent the highest classification accuracy in each column, and underlined values represent the second-highest classification accuracy in each column.

### 4.4 Visualization

To further evaluate the performance of our model, we employed t-SNE (Li et al., 2016) as a data visualization technique. T-SNE is widely used in machine learning tasks for dimensionality reduction, particularly to visualize high-dimensional data such as images, text, and feature representations. The T-SNE visualization of the proposed method PGCANet is illustrated in Figure 6. It is evident that, after processing by the PGCANet model, the boundaries between different categories are more distinct, demonstrating that the model effectively performs classification. We note that t-SNE 2D projection may produce misleading visual clusters dominated by subject-specific variations. Therefore, our interpretation is not solely based on qualitative visualization. The consistent high classification accuracy reported in Section 4.3 provides quantitative evidence that the model learns task-discriminative features instead of subject-related patterns.

**Figure 6:**
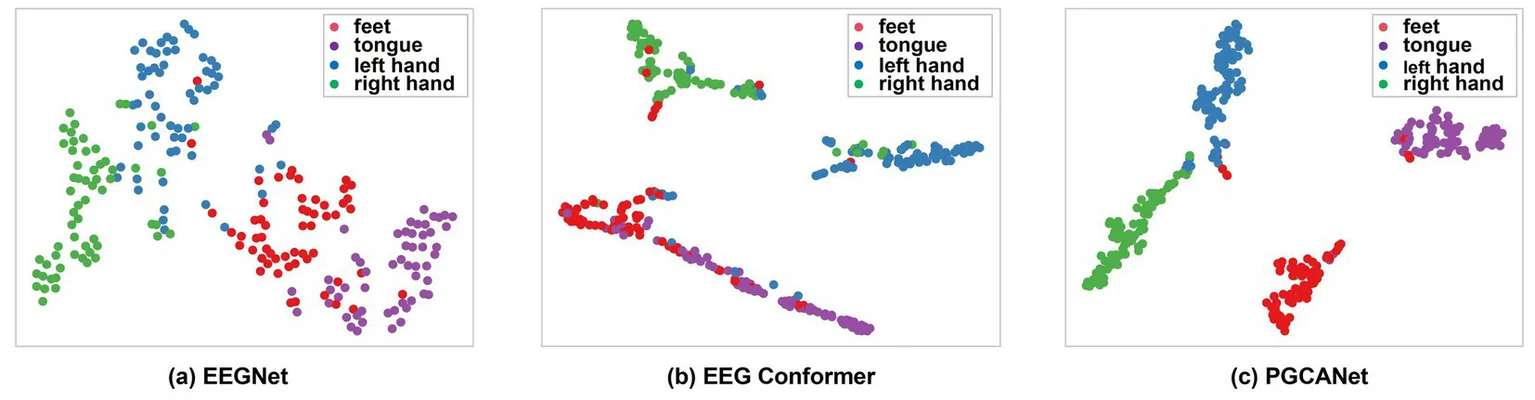
The t-SNE visualization on the BCI IV-2A dataset compares the performance of the different models for quadruple classification. Different colors represent different categories.

### 4.5 Ablation experiment

We conducted ablation experiments on BCI IV-2A to evaluate three components: the channel-aware module, the time-frequency branch, and the consistency-constrained loss. As shown in Table 5, the model achieves the highest accuracy of 82.47% when all three components are enabled. Removing any component reduces classification accuracy, and the largest performance drop occurs when the consistency-constrained loss is discarded. Among the three modules, the consistency-constrained loss brings the largest performance gain, followed by the time-frequency branch and the channel-aware module. These results confirm the effectiveness of each component, verify their positive individual contributions to decoding performance, and demonstrate that joint cooperation of all three components yields optimal accuracy.

**Table 5:** Ablation experiments of different modules on the BCI IV-2A dataset.

Chan-aware	Time-fre	Cc-loss	Avg. ACC
			78.66
√			79.35
	√		80.20
		√	79.88
√	√		80.65
√		√	80.94
	√	√	80.86
√	√	√	82.47

The adjustable parameter *λ* in the consistency-constrained fusion loss is empirically determined. The results presented in Table 6. As a balancing coefficient for training consistency, with its optimal value ensuring the loss terms’ contribution ratio fits the task. Ablation experiments confirm *λ* = 0.02 is its optimal value, providing quantitative guidance for experimental parameter selection and reducing reliance on subjective empirical estimates.

**Table 6:** Ablation experiment on λ value selection on the BCI IV-2A dataset.

λ = λ_1_ = λ_2_	S01	S02	S03	S04	S05	S06	S07	S08	S09	Avg	Std
λ = 0.5	75.86	55.17	75.00	49.14	68.97	53.45	57.76	87.07	70.69	65.90	11.97
λ = 0.2	81.03	56.90	79.31	52.59	48.28	52.59	71.55	90.52	77.58	67.82	14.51
λ = 0.1	83.62	70.69	93.10	62.07	75	60.34	75.00	94.83	90.52	78.35	12.18
λ = 0.09	89.66	38.79	95.69	68.97	52.59	61.21	69.83	93.10	88.79	73.18	18.85
λ = 0.08	90.52	69.83	95.69	68.10	58.62	58.62	74.14	93.97	87.07	77.40	13.88
λ = 0.07	92.24	61.21	93.97	67.24	50.86	66.38	75.00	93.97	89.66	76.72	13.88
λ = 0.06	90.52	56.03	95.67	68.10	78.45	73.28	75.00	93.10	87.93	79.79	12.37
λ = 0.05	92.24	70.69	94.83	72.41	70.69	67.24	68.97	92.24	92.24	80.17	11.47
λ = 0.04	89.66	68.10	95.69	68.10	62.93	68.10	94.83	93.10	89.66	81.23	12.94
λ = 0.03	91.38	56.90	96.55	70.69	78.45	68.97	94.83	95.69	87.93	82.38	13.44
**λ = 0.02**	92.24	63.79	93.97	68.97	82.76	64.66	93.10	91.38	91.38	**82.47**	12.24
λ = 0.01	89.66	62.93	94.83	78.45	70.69	63.79	95.69	94.83	89.66	82.28	12.82
λ = 0.009	92.24	65.52	95.69	76.72	41.38	67.24	95.69	93.10	90.52	79.79	17.63

Bold values represent the highest classification accuracy in each column, and underlined values represent the second-highest classification accuracy in each column.

## 5 Discussion

To evaluate the concentration level of the subjects during MI and track the continuous brain electrical feature changes from imagery to execution, this paper proposes for the first time a novel cue-execution two-stage MI paradigm CEP-MI and analyzes the neural response characteristics of different limbs during MI using ERP and ERS/ERD methods. Further, a dual-branch decoding architecture combining prior knowledge with channel-aware feature learning is proposed. Our findings are as follows: (1) ERP analysis revealed that the cue-execution MI task elicited lower P3 amplitude compared to the classic task. Specifically, for right-hand movement imagination, the cue-execution MI task resulted in a lower LPC amplitude than the classic MI task. (2) Time-frequency analysis demonstrated typical lateralized patterns during hand MI, with contralateral ERD and ipsilateral ERS in the *μ* rhythm. (3) The proposed model achieved accuracies of 85.77 and 80.82% in distinguishing between left and right hands in the cue-execution MI task and the classic MI task, respectively; and accuracies of 82.06 and 80.00% in distinguishing between left and right feet in the cue-execution MI task and the classic MI task, respectively. ME improved the discriminability of limb-specific intentions.

### 5.1 ERP results analysis

P300 is primarily linked to parietal and frontal regions responsible for attention allocation and decision-making (Carrillo-de-la-Peña and Cadaveira, 2000). During MI, the cognitive network underlying P300 participates in regulation, and decision processes in the frontal lobe can elicit the P300 component (Tomeh et al., 2022). In our data, the classic MI task evokes higher P300 amplitudes than cue-execution MI task. This likely reflects a greater emphasis on stimulus evaluation and decision-making in the classic MI condition. When execution demands are added, neural resources partially shift toward motor preparation and control, leading to a relative reduction of parietal P300 (Ronca et al., 2026; Guerrero et al., 2022).

The LPC is closely associated with cognitive integration and sustained attentional allocation, and it often indexes reasoning or task-rule integration (Lin et al., 2022). In the classic MI task, the right hand elicits a stronger LPC than the left hand, suggesting lateralization that may relate to participants’ handedness. The LPC amplitude is also higher in the classic MI condition than in the execution condition, indicating that late-stage cognitive integration and evaluation yield to motor preparation and inhibitory control when execution is introduced. The coordinated changes in P300 and LPC jointly support a neural transition from MI to ME.

It should be noted that the reduced P300 and LPC amplitudes may also arise from alternative factors. The CEP-MI paradigm incorporates motor execution, which increases task complexity and may lead to the dispersion of attentional resources. ERP components originate from the superposition of multiple neural sources and do not map uniquely to a single cognitive process. Nevertheless, combined with the time-frequency ERD/ERS evidence obtained in this study, we propose that the reallocation of cognitive resources from stimulus evaluation toward motor preparation serves as the dominant underlying mechanism, while increased task complexity and divided attention may serve as contributing factors.

### 5.2 Cross-task time-frequency analysis

Through time-frequency analysis, we examined ERS and ERD within the 8–30 Hz range during MI tasks. We observed significant *μ*/*β* ERD at C3 and C4 during classic hand MI, consistent with the core physiological mechanism of MI, in which the contralateral sensorimotor cortex is activated (Mitsuhashi et al., 2024). We also noted sustained activation of the motor cortex during continuous imagery. When MI incorporated ME, the ERD amplitude decreased, and a distinct ERS peak emerged at approximately 2–3 s. This pattern likely reflects a shift from imagery-related activation to a motorpreparatory state. At that stage, the basal ganglia initiate inhibitory control of the motor cortex to prevent excessive excitation carried over from the imagery phase, which could otherwise precipitate movement disorders. These findings indicate active cortical regulation during the transition from imagery to execution (Dekleva et al., 2024).

In the classic foot MI task, overall ERD and ERS showed minimal temporal variation. And the curves of the left and right feet have a high degree of overlap, lacking obvious stage-specific distinction. This suggests smooth, sustained activation of the motor cortex during prolonged foot imagery. The observation is consistent with prior studies locating the foot motor area near Cz along the midline (McDonald et al., 2025; Quadrelli et al., 2019). After introducing ME, the curves diverged markedly in the final 2–3 s of the imagery phase. This indicates that actual execution enhances the brain’s ability to differentiate movement intentions between the left and right limbs. Collectively, these results provide direct physiological evidence for the neural transition from MI to ME (Pei and Vinjamuri, 2025).

### 5.3 Analysis of classification results

The classification experiment results demonstrate that PGCANet outperforms several advanced methods in the field. It achieves an accuracy of 82.47% on the four-class classification task using the BCI IV-2A dataset. On the CEP-MI dataset, PGCANet attains binary classification accuracies of 80 and 82.06% for the two respective tasks. Compared to the baseline model, which focuses solely on spatiotemporal feature extraction, PGCANet improves accuracy by 3%. Additionally, it significantly outperforms multi-domain feature fusion algorithms such as M-FANet (Qin et al., 2024) and FBCNet (Mane et al., 2021). Most existing multi-branch decoding approaches for MI-EEG rely on simple feature concatenation for fusion. By contrast, our joint-training strategy with consistency constraints can exploit complementary information across branches and mitigate noise interference from individual branches, yielding more robust representations of motor intention. Ablation experiments further confirm that the prior guided time-frequency feature extraction branch enhances the model’s classification performance by 1.53%. These findings not only validate the rationale behind PGCANet architecture and training strategy but also provide valuable insights for parameter optimization and performance tuning in future models.

In addition to quantitative performance changes observed in the ablation experiments, several methodological implications can be drawn. The consistency-constrained fusion loss brings the largest performance gain, indicating that cross-branch collaborative regularization is critical for multi-domain EEG decoding. Simply concatenating features from different branches cannot fully exploit complementary information; instead, consistency-driven joint training helps the model learn stable and robust task-related representations. Moreover, both the channel-aware module and time-frequency branch contribute positively to decoding, which demonstrates that combining spatial-domain adaptive channel weighting with prior-guided time-frequency feature extraction is an effective strategy for MI-EEG modeling. These findings provide practical guidance for designing dual-branch MI-BCI decoding architectures in future studies.

Binary classification experiments on the CEP-MI dataset corroborate the earlier ERP and time-frequency analysis results. First, upper limb MI consistently achieved higher accuracy than lower limb MI across both tasks. This aligns with the pronounced lateralization effect observed in upper limb MI. It also explains the relatively poorer classification performance for lower limb MI, due to the proximity of its control centers to the midline Cz electrode. Second, cue-execution MI task consistently produced superior classification results compared to classic MI task in both upper and lower limb MI classifications. This suggests that incorporating actual ME enhances the brain’s ability to distinguish between left- and right-limb movement intentions.

We further analyze the bilateral differences between left-hand and right-hand motor imagery in terms of classification performance and feature visualization results. Experimental results indicate that the two imagery directions present asymmetric behavioral and feature patterns. Such bilateral differences are consistent with the inherent functional lateralization of the human sensorimotor cortex, where left- and right-limb imagery induces activation of distinct contralateral cortical regions with varying activation intensity and stability. Correspondingly, the t-SNE visualization also reveals inconsistent cluster separation degrees between the two categories. These asymmetric phenomena demonstrate that left-side and right-side motor imagery tasks yield distinguishable neural response characteristics, leading to naturally different feature distributions and classification performances across the two tasks.

Despite the advancements discussed above, this study has several limitations. First, it focuses solely on investigating the neural mechanisms underlying MI for hand and foot limb movements. Given PGCANet’s sensitivity to spatial and frequency patterns, future research should explore its performance in other BCI paradigms, such as the P3 spelling task. Second, recent studies have demonstrated single-finger control of robotic hands by integrating EEG with deep learning (Ding et al., 2025). Therefore, future work should utilize existing finger-level fine motor data to develop efficient EEG decoding frameworks for fine motor control. It should be noted that all analyses in the present study are based on offline data collected from healthy participants. The conclusions drawn are limited to offline-decoding performance. Further online BCI validation is required before we can claim practical advantages for real-time applications.

## 6 Conclusion

This study introduces an MI task that integrates ME, which is referred to as CEP-MI. This approach elucidates the neural mechanisms involved in the transition from MI to ME. Meanwhile, it suggests that the incorporation of ME improves the classification performance of MI. Additionally, this study presents a MI decoding architecture named PGCANet, which avoids the dilution of core time-frequency features caused by full-electrode processing and addresses the limitation of insufficient focus on spatial information in traditional spatiotemporal convolutions. We conducted extensive experiments on two datasets, and the results indicate that our method compares favorably with other comparative methods. Due to the improvement in classification performance, CEP-MI offers new insights into the MI experimental paradigm. PGCANet shows favorable decoding performance and can offer references for related BCI research.

## Data Availability

The raw data supporting the conclusions of this article will be made available by the authors, without undue reservation.

## Footnotes

1
https://sccn.ucsd.edu/wiki/EEGLAB_Extensions
